# Dual targeting of RdRps of SARS-CoV-2 and the mucormycosis-causing fungus: an *in silico* perspective

**DOI:** 10.2217/fmb-2022-0083

**Published:** 2022-05-05

**Authors:** Abdo A Elfiky

**Affiliations:** ^1^Biophysics Department, Faculty of Sciences, Cairo University, Giza, 12613, Egypt

**Keywords:** computational drug design, drug repurposing, mucormycosis, nucleotide inhibitors, RdRp, SARS-CoV-2

## Abstract

During the past few months, mucormycosis has been associated with SARS-CoV-2 infections. Molecular docking combined with molecular dynamics simulation is utilized to test nucleotide-based inhibitors against the RdRps of SARS-CoV-2 solved structure and *Rhizopus* *oryzae* RdRp model built *in silico*. The results reveal a comparable binding affinity of sofosbuvir, galidesivir, ribavirin and remdesivir compared with the physiological nucleotide triphosphates against *R.* *oryzae* RdRp as well as the SARS-CoV-2 RdRp as reported before. Additionally, other compounds such as setrobuvir, YAK, IDX-184 and modified GTP compounds 2, 3 and 4 show potential calculated average binding affinities against *R. oryzae* RdRp. The present *in silico* study suggests the dual inhibition potential of the recommended drugs and compounds against SARS-CoV-2 and *R.* *oryzae* RdRps.

SARS-CoV-2 and its associated pneumonia, COVID-19, bestowed a heavy health and economic burden during the 2 years after its initial outbreak [1–3]. Furthermore, the currently developed pandemic of mucormycosis in COVID-19 patients and COVID-19 recovered people has caused another health debate about the therapeutics approved for COVID-19 [4,5]. The deadly mucormycosis fungal infection is a rare disease that has developed in patients suffering from reduced immunity conditions such as during organ transplants, cancer patients and diabetic patients [6]. The leading cause of mucormycosis is the *Rhizopus* *oryzae* fungus [7]. RNA-dependent RNA polymerase (RdRp) is a crucial pathogenic replicase protein usually targeted [8–10].

Nucleotide inhibitors are potential antiviral polymerases (RdRps) and are successful candidates for targeting and inhibiting a different group of viral polymerases. We previously reported the possible binding affinities of some nucleotide inhibitors against SARS-CoV-2 RdRp models and solved structures [11]. Additionally, some modified guanosine derivatives are suggested by our group to be potential candidates against HCV and ZIKV [12].

Several *in silico* studies can suggest possible therapeutics against COVID-19 even before depositing any solved structures for the viral proteins. Some of the recommended drugs are now approved by the US FDA and save lives [13,14].

In this study, our strategy is to test different groups of nucleotide inhibitors against both RdRps of SARS-CoV-2 and *R.* *oryzae* to find the possible dual effective drug (repurposing) or drug-like compounds. Molecular docking combined with molecular dynamics simulation (MDS) is used in the current study to test the binding against different fungal RdRp conformations. To the best of our knowledge, this study reports for the first time the effectiveness of FDA-approved drugs (e.g., galidesivir, ribavirin, remdesivir) against the RdRp of *R.* *oryzae*. These drugs were approved before against different viruses, but we hypothesize that they can be repurposed in mucormycosis. Additionally, novel guanosine derivatives are introduced to have an anti-RdRp effect for *R.* *oryzae* and SARS-CoV-2. Therefore, we suggest experimentally testing these drugs and the new guanosine derivatives to judge our computational findings.

## Materials & methods

### Structural preparations

The ligands' structures were prepared in their active (triphosphate) form with GaussView 5 software. Geometry optimization was performed on the ligands with the aid of the classical Universal Force Field (UFF), then semi-empirical parameterization method 6 (PM6) was applied [15–17]. Cinnamaldehyde was used as a negative control compound, whereas GTP, UTP, ATP and CTP were tested as positive controls. These nucleotides are the physiological molecules that fit inside the active site of the RdRp, and we need to compete with them for the RdRp active site. However, cinnamaldehyde was reported to be of low affinity to the active site of RdRp.

The 3D structure of the RdRp from *R.* *oryzae* was built using the SWISS-MODEL web server. It was then validated using the Molproperty server and Structure Analysis and Verification Server (SAVES) [18]. PyMOL and AutoDock Tools software were utilized to prepare the protein structures for the docking after the MDS run [19–21]. Charges and missing hydrogen atoms were added to the RdRp model before the MDS by AutoDock Tools.

### Molecular dynamics simulation

The *R.* *oryzae* RdRp model was subjected to a 100-ns MDS run at the normal volume and temperature (NVT) ensemble condition. NAnoscale Molecular Dynamics (NAMD) 2.13 software was used to run the MDS. Visualizing Molecular Dynamics (VMD) 1.9.3 software was utilized to prepare the system before the simulation and analyze the data after the MDS run [22,23]. The protein was first solvated using the TIP3P water model, and NaCl was added to the solution with a concentration of 0.154 M at the physiological temperature of 37°C [24]. The protein–water system was minimized for 10,000 steps of steepest descent before the MDS run. The used force field during the simulation was CHARMM 36 [24,25]. After the production run, cluster analysis of the trajectories was performed using the USCF Chimera 1.14 software [26]. Representative conformations of the protein from the seven most populated clusters were used in the docking experiments.

### Molecular docking

A 24-core HP Z8 workstation was utilized for the docking calculations using AutoDock Vina software [20]. Every molecule was docked into the seven conformations of the RdRp model, where average values were calculated and are presented in the Results section. All docked experiments maintained a flexible ligand in a flexible active site (D193 and D194) protocol. The grid box used in the calculations of the binding energies was set to 30 × 30 × 30 Å^3^ in size to cover the active site residues. The search box was centered at the active site with coordinates around (-8, -18, 30). Finally, the docking complexes were examined using Discovery Studio Visualizer v21.1 [27].

## Results

The SWISS-MODEL webserver was utilized for building the RdRp model of *R.* *oryzae*. First, we got the sequence from the National Center for Biotechnology Information (NCBI) protein database (GenBank: BAH03542.1), then we searched for a template to build our model. We used the template structure 7KFT chain D, as it possesses the highest coverage (0.82) and 15.02% sequence identity to our query sequence. The model was valid based on SWISS-MODEL validation parameters: GMQE (0.41) and QMEANDisCo global score (0.41 ± 0.05). We also validated the model using the Molprobity web server, where the Ramachandran plot (Supplementary Figure 1) gives 87.2% in the most favored regions and 94.0% in the allowed regions. This model was energy minimized in 10,000 steps of the steepest descent algorithm before the MDS run.

**Figure 1.**
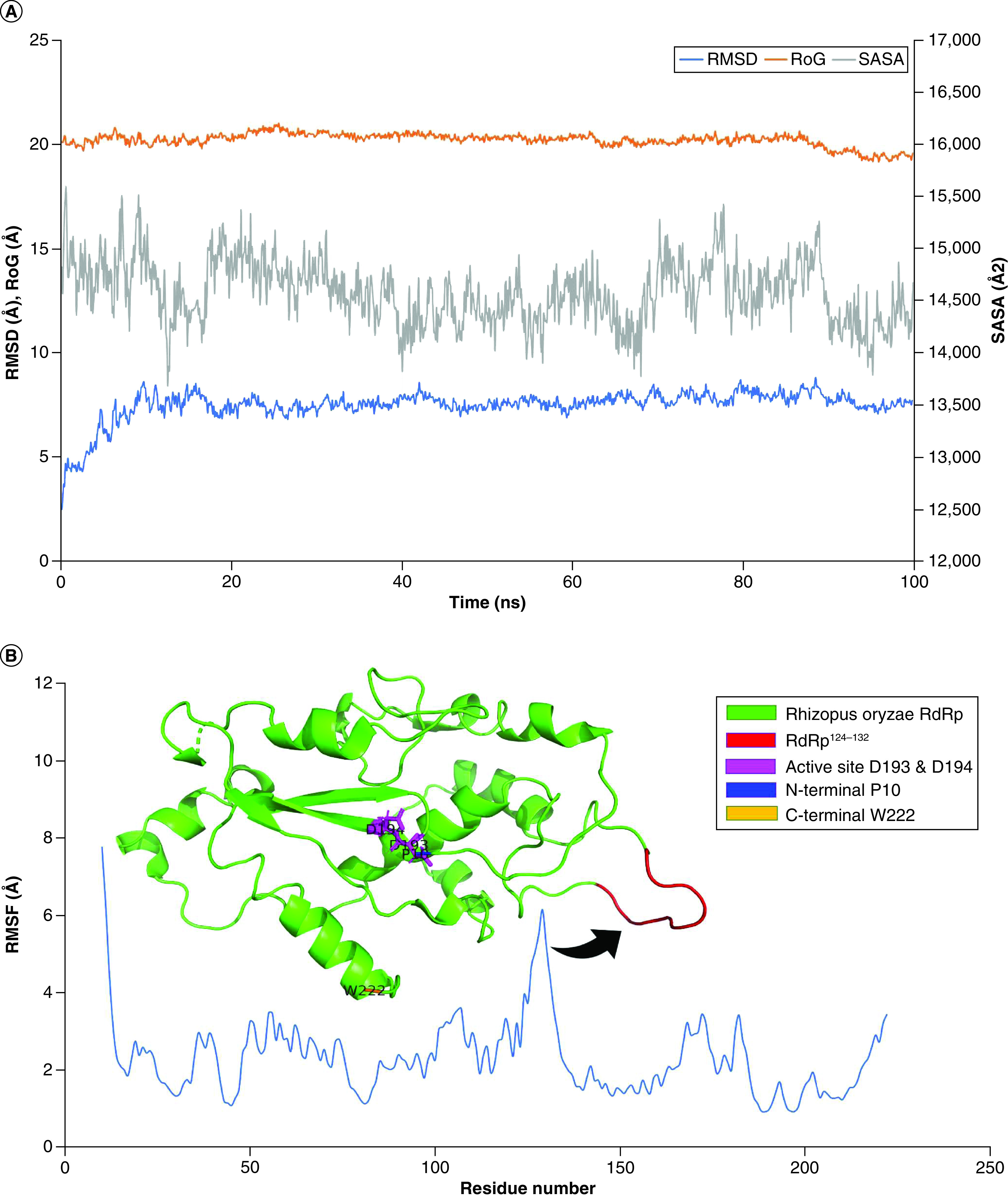
Molecular dynamics simulation analysis. **(A)** The root mean square deviation (RMSD) in Å (blue line), radius of gyration (RoG) in Å (orange line) and solvent-accessible surface area (SASA) in Å^2^ (gray line) versus the simulation time in nanoseconds. **(B)** The per-residue root mean square fluctuation (RMSF) in Å. The protein structure is represented in colored cartoons as per the legend coloring scheme.

The dynamics of the *R.* *oryzae* RdRp model are shown in Figure 1. This study aims to test some potential RdRp inhibitors against the *R.* *oryzae* RdRp using the all atoms 3D model equilibrated through MDS for up to 100 ns. We decomposed the results into three categories: the binding affinity of some FDA-approved drugs, modified guanosine derivatives and some different compounds that showed promising results previously against other pathogenic infections.

Figure 1A shows the root mean square deviation (RMSD; blue line), radius of gyration (RoG; orange line), and solvent-accessible surface area (SASA; gray line) variation with the simulation time (100 ns). The system was equilibrated within the first 20 ns of the simulation with RMSD around 7.5Å. The RoG and SASA values were stable (20Å and 14,500Å^2^, respectively) during the entire simulation period. Figure 1B shows the per-residue RMSF during the whole period of the simulation (blue line). The highly fluctuating regions are depicted in a colored cartoon in the upper part of the figure, whereas other protein regions are in green cartoons. For example, the L124-A132 region is red, whereas the N-terminal (P10) and C-terminal (W222) are depicted in blue and orange cartoons, respectively. The active site aspartates (D193 and D194) are shown in magenta sticks.

### Binding affinities of the anti-RdRp drugs against *R. oryzae* RdRp

**Figure 2.**
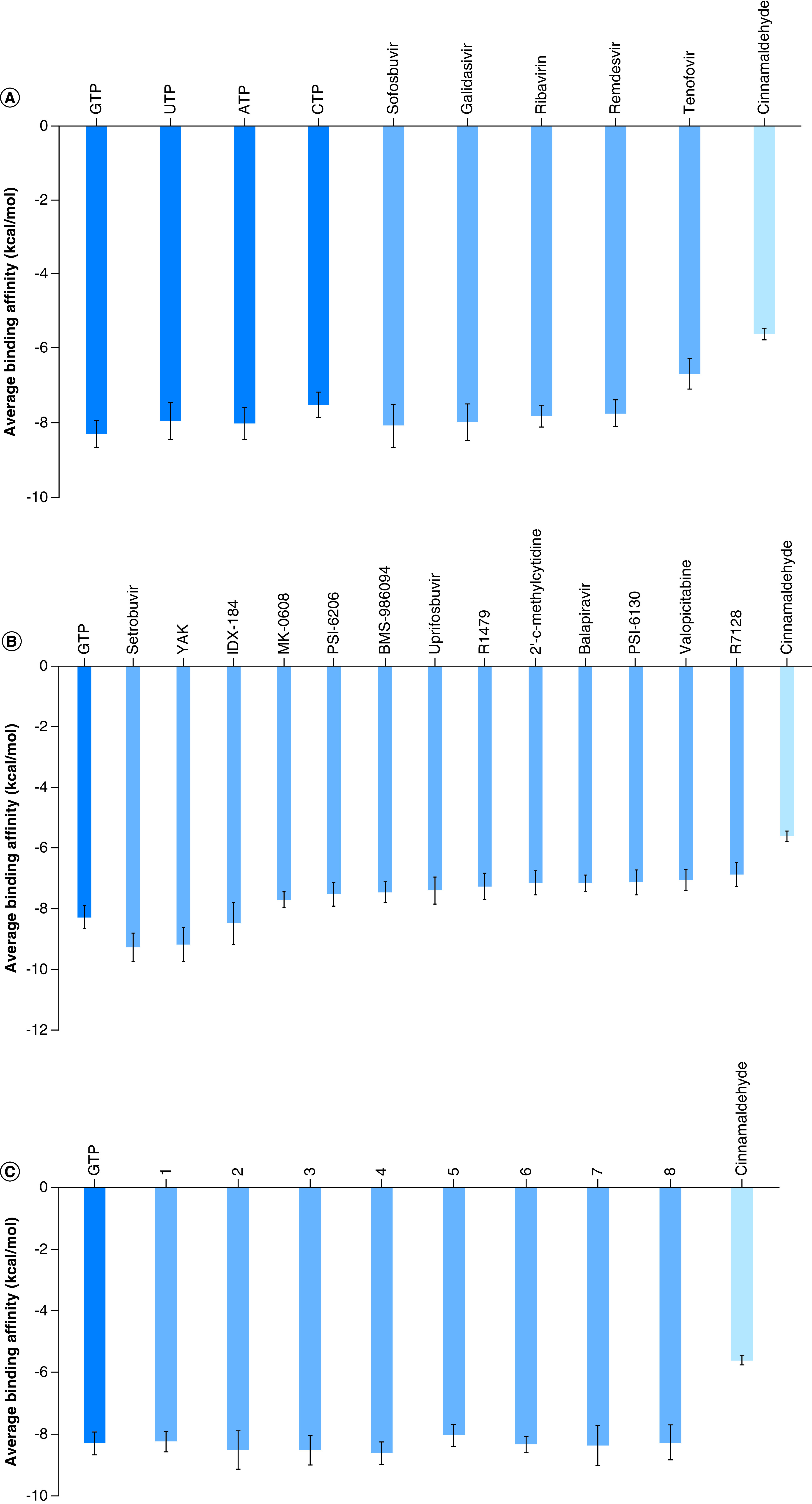
Binding affinity calculations. The average binding affinities (in kcal/mol) for **(A)** the drugs sofosbuvir, galidisivir, ribavirin, remdesivir and tenofovir along with the physiological nucleotides GTP, UTP, ATP and CTP. **(B)** Setrobuvir, YAK, IDX-184, MK-0608, PSI-6206, BMS-986094, uprifosbuvir, R1479, 2′ C-methylcytidine, balapiravir, PSI-6130, valopectipine and R7128 along with the physiological nucleotide GTP and the negative control cinnamaldehyde. **(C)** The modified GTP derivative compounds 1-8. The docking calculations were performed on seven different protein conformations after cluster analysis of the 100-ns MDS trajectories. Then the average value was plotted with error bars to represent the standard deviation.

Figure 2A shows the average binding energies (in kcal/mol) for sofosbuvir, galidisivir, ribavirin, remdesivir and tenofovir (blue columns) in addition to the positive controls GTP, UTP, ATP and CTP (green columns) and the negative control cinnamaldehyde (red column). The average binding energies for the tested drugs lie between -8.10 and -6.70 kcal/mol, whereas for the nucleotides, the average binding energies are between -8.30 and 7.53 kcal/mol.

### Binding affinities of some potential compounds against *R. oryzae* RdRp

Figure 2B shows the average binding energies for *R.* *oryzae* RdRp against setrobuvir, PAEBVIJMNXTTAT_AEFFLSMTSA_N (YAK), IDX-184, MK-0608, PSI-6206, BMS-986094, uprifosbuvir, R1479, 2′ C-methylcytidine, balapiravir, PSI-6130, valopectibine and R7128 (blue columns). Additionally, the parent compound GTP (green column) and the negative control cinnamaldehyde (red column) are shown for comparison. The first three compounds (setrobuvir, YAK and IDX-184) show better average binding affinities (-9.27 ± 0.47, -9.20 ± 0.56 and -8.47 ± 0.70 kcal/mol, respectively) against the RdRp active site compared with the parent compound (GTP -8.30 ± 0.37 kcal/mol). In contrast, the other compounds show slightly higher average binding energies (between -7.7 and -6.87 kcal/mol), but are still better than the cinnamaldehyde (-5.61 ± 0.16 kcal/mol).

### Binding affinities of modified guanosine derivatives against *R. oryzae* RdRp

Figure 2C shows the average binding energies for *R.* *oryzae* RdRp against eight modified guanosine triphosphate derivatives (blue columns). The hydroxyl group at position 2′ is replaced by 2,6-dihydroxyphenyl oxidanyl (1), 2-hydroxyphenyl oxidanyl (2), 3,5-dihydroxyphenyl oxidanyl (3), 3-hydroxyphenyl oxidanyl (4), 3,5-disulfanylphenyl oxidanyl (5), 3-sulfanylphenyl oxidanyl (6), 3-fluorophenyl oxidanyl (7) and 4-fluorophenyl oxidanyl (8). GTP (green column) and cinnamaldehyde (red column) are also shown for comparison.

### Binding modes of the tested drugs & compounds against *R. oryzae* RdRp

**Figure 3.**
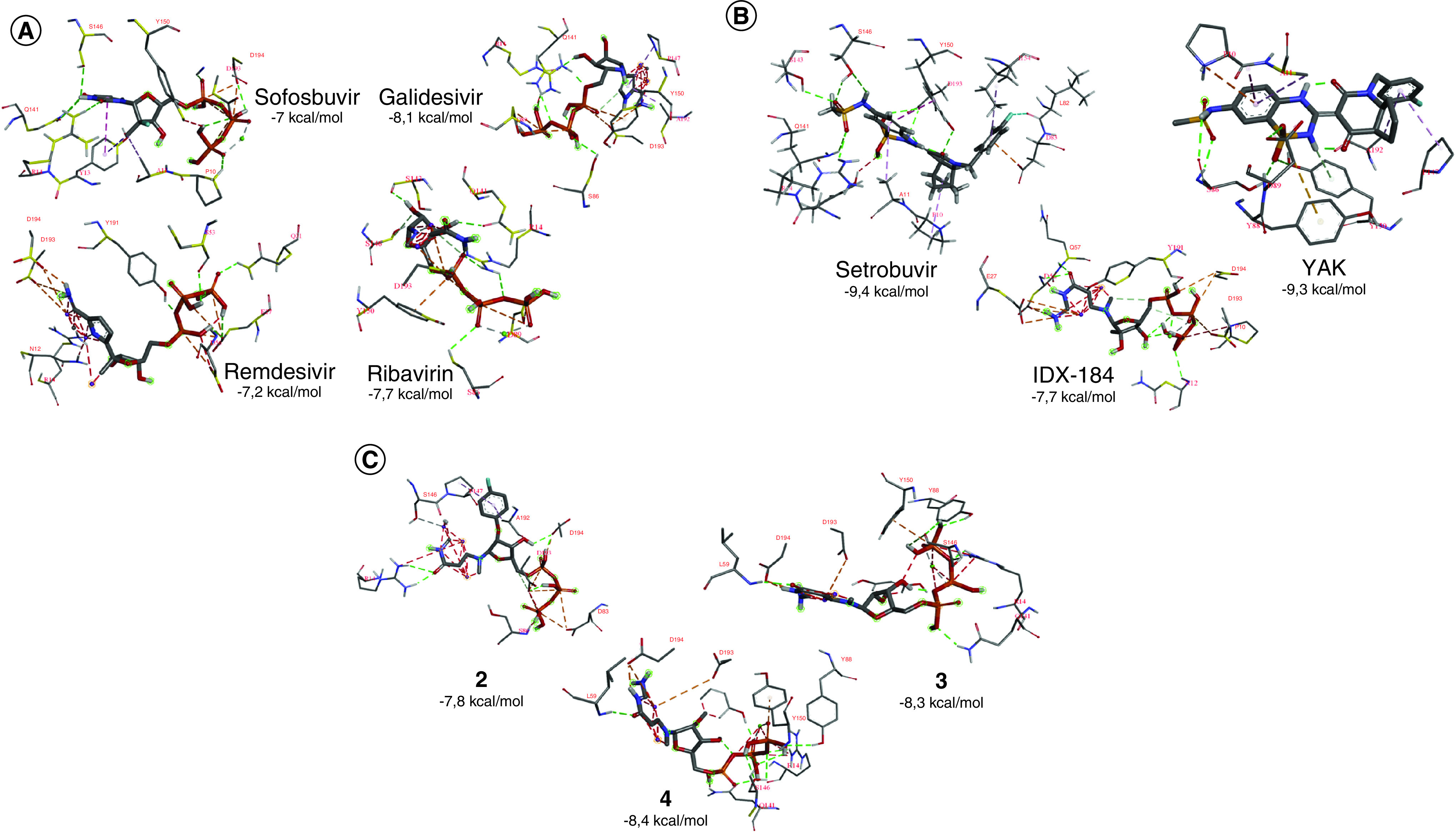
Docking poses. The interaction patterns for **(A)** the best four drugs sofosbuvir, galidisivir, ribavirin and remdesivir, **(B)** setrobuvir, YAK and IDX-184, and **(C)** GTP derivative compounds 2, 3 and 4, after docking into the active site of *Rhizopus oryzae* RdRp. The residues from *R. oryzae* RdRp that form contacts with the drugs are depicted in lines, whereas the drugs are in a stick. H-bonds are shown in green dashed lines, whereas hydrophobic interactions are in gray dashed lines. Halogen bonds are shown in cyan dashed lines, whereas π-π stacking, π-anion and π-alkyl are in violet, orange and magenta dashed lines, respectively.

The aid of Discovery Studio Visualizer assesses the interactions established upon docking the drugs sofosbuvir, galidisivir, ribavirin and remdesivir into the active site of *R.* *oryzae* RdRp. Figure 3A shows the formed interactions with the coloring scheme as mentioned in the figure legend. The interactions between the best three compounds, setrobuvir, YAK and IDX-184, are represented in Figure 3B. However, Figure 3C shows the formed interactions in the best three guanosine derivatives, compounds 2, 3 and 4. Table 1 summarizes the tested drugs and compounds’ formed interactions in selected complexes. Bold residues are the active site aspartates D193 and D194, whereas underlined residues are the most reported interactions.

**Table 1.** The interactions formed between some nucleotide inhibitors and the *Rhizopus oryzae* RdRp upon docking.

Compound	AutoDock score (kcal/mol)	H-bonding		Hydrophobic interaction	
		Number	Amino acids involved	Number	Amino acids involved
GTP	‐8.0	8	P10, D56, G142, S143, **D193** and **D194(3)**	4	E27(2), D56 and S143
Sofosbuvir	‐7.0	11	P10, R14, Q141(2), S146, **D193(3)** and **D194(3)**	4	A11, Y13(2) and Y150
Galidasivir	‐8.1	13	R14(3), S86, D89(3), Q141, **D193(3)** and **D194(2)**	3	P147, Y150 and A192
Ribavirin	‐7.7	13	R14(3), S86, D89(2), Q141, S143, S146 and **D193(4)**	1	Y150
Remdesivir	‐7.2	11	E27(2), Q31, S53, D56(2), Y191, **D193(2)** and **D194(2)**	5	N12, R14 and E27(3)
Setrobuvir	‐9.4	9	R74, L82, Q141, S143, S146(2), Y150 and **D193(2)**	5	P10, A11, H54, D83 and Y150
YAK	‐9.3	5	S86(3), D89 and Y150	7	P10(2), A11, Y88, P147, Y150 and A192
IDX-184	‐7.7	10	N12, E27(2), D56(2), Q57, Y191, **D193** and **D194(2)**	1	P10
2	‐7.8	12	R14(2), D83(2), **D193(5)** and **D194(3)**	3	S86, P147 and A192
3	‐8.3	10	R14, L59, Y88, Q141, S146(3), **D193** and **D194(2)**	1	R14
4	‐8.4	10	R14, L59, Y88, S146(4), **D193** and **D194(2)**	2	Q141 and Y150

## Discussion

Anti-RdRp drugs are a successful group against different pathogens, including viruses and fungal infections. Many FDA-approved anti-RdRps can show potential inhibition of many infectious diseases due to their high safety and specificity against pathogens’ RNA. From Figure 2A, sofosbuvir, galidisivir, ribavirin and remdesivir show comparable average binding affinities to the nucleotide triphosphate. These drugs gave the same trend as possible therapeutic options against COVID-19, with remdesivir approved by the FDA for emergency use [28–30]. These drugs have been tested for other viruses and have knowable safety profiles in millions of people, including HCV survivors.

However, setrobuvir, YAK and IDX-184 show better (lower) average binding energy against the *R. oryzae* RdRp active site than the GTP. Setrobuvir has an 11.7% reduction in the binding energy compared with GTP, whereas YAK and IDX-184 have 10.8% and 2% reductions, respectively. Setrobuvir was in clinical trials against HCV NS5b in combination with ribavirin and with or without interferon [31]. Setrobuvir, YAK and IDX-184 show very high binding potentials to RdRps of HCV, ZIKV and SARS-CoV-2. Still, experimental validation is yet to be conducted. The eight modified GTP derivatives show comparable average binding affinities to the *R.* *oryzae* RdRp active site to that of the physiological parent compound GTP. The best three compounds, 2, 3 and 4, are shown in Figure 3C, and the formed interactions are listed in Table 1. Again, experimental safety testing and validation are required for these modified GTP compounds.

The main driving force for drug binding was the formation of H-bonds. Few hydrophobic interactions (π-anion, π-alkyl, π-π stacking and alkyl interactions) and halogen interactions are formed as well in setrobuvir. The active site residues D193 and D194 are critical for binding drugs and compounds, whereas YAK is an exception. The two active site aspartates form H-bonding and charge interactions with the drugs and compounds. The drugs sofosbuvir, galidisivir, ribavirin and remdesivir create an average of 12 H-bonds, 40% with D193 and D194. This is also observed in GTP (50%) and the modified GTP compounds (44%). However, setrobuvir and IDX-184 have lower contributions of the H-bonds formed with the active site aspartates (26%), whereas YAK has neither H-bonds created with D193 nor D194. The residue Y150 develops an H-bond to only YAK, whereas it is involved in hydrophobic interaction (π-π stacking) with sofosbuvir, galidasivir, ribavirin, setrobuvir, YAK and the GTP modified compound 4. Additionally, the residue P10 forms alkyl interaction with setrobuvir, YAK and IDX-184, whereas it forms an H-bond with GTP. However, the residue R14 forms both H-bonds (sofosbuvir, galidisivir, ribavirin, and the compounds 2, 3 and 4) and hydrophobic interactions (remdesivir and compound 3) upon docking. Additionally, Q141 forms H-bonds with sofosbuvir, galidisivir, ribavirin, setrobuvir and compound 3, forming hydrophobic contact with compound 4.

## Conclusion

Nucleotide inhibitors displayed an excellent outcome in their efficacy against many viruses, including SARS-CoV-2. Sofosbuvir, galidisivir, ribavirin and remdesivir showed comparable calculated average binding affinity against the RdRp of *R.* *oryzae*, the leading cause of mucormycosis. Additionally, other compounds such as setrobuvir, YAK, IDX-184 and some modified GTP compounds (2, 3 and 4) showed better or comparable average binding affinity against *R.* *oryzae* RdRp *in silico*. These drugs and compounds have proved their potency against SARS-CoV-2 and could be effective against mucormycosis. Further, experimental validation is highly recommended for the suggested drugs and modified compounds.

**Summary points**

- Mucormycosis has been reported associated with SARS-CoV-2 infections in India and other countries.
- The RdRp of *Rhizopus* *oryzae* has two consecutive aspartates, a hallmark of viral RdRp.
- Sofosbuvir, galidesivir, ribavirin and remdesivir have excellent binding affinity against *R.* *oryzae* and SARS-CoV-2 RdRps *in silico*.
- Setrobuvir, YAK, IDX-184, and modified GTP compounds 2, 3 and 4 show potential calculated average binding affinities against *R.* *oryzae* RdRp.
