# Supplementary material for: Dual targeting of RdRps of SARS-CoV-2 and the mucormycosis-causing fungus: an in silico perspective
Source: Future Microbiol. 2022 May 5:10.2217/fmb-2022-0083. doi: 10.2217/fmb-2022-0083 (PMC9070561; doi:10.2217/fmb-2022-0083)
Supplement: Supplementary file 1 [file Figure-S1.docx]

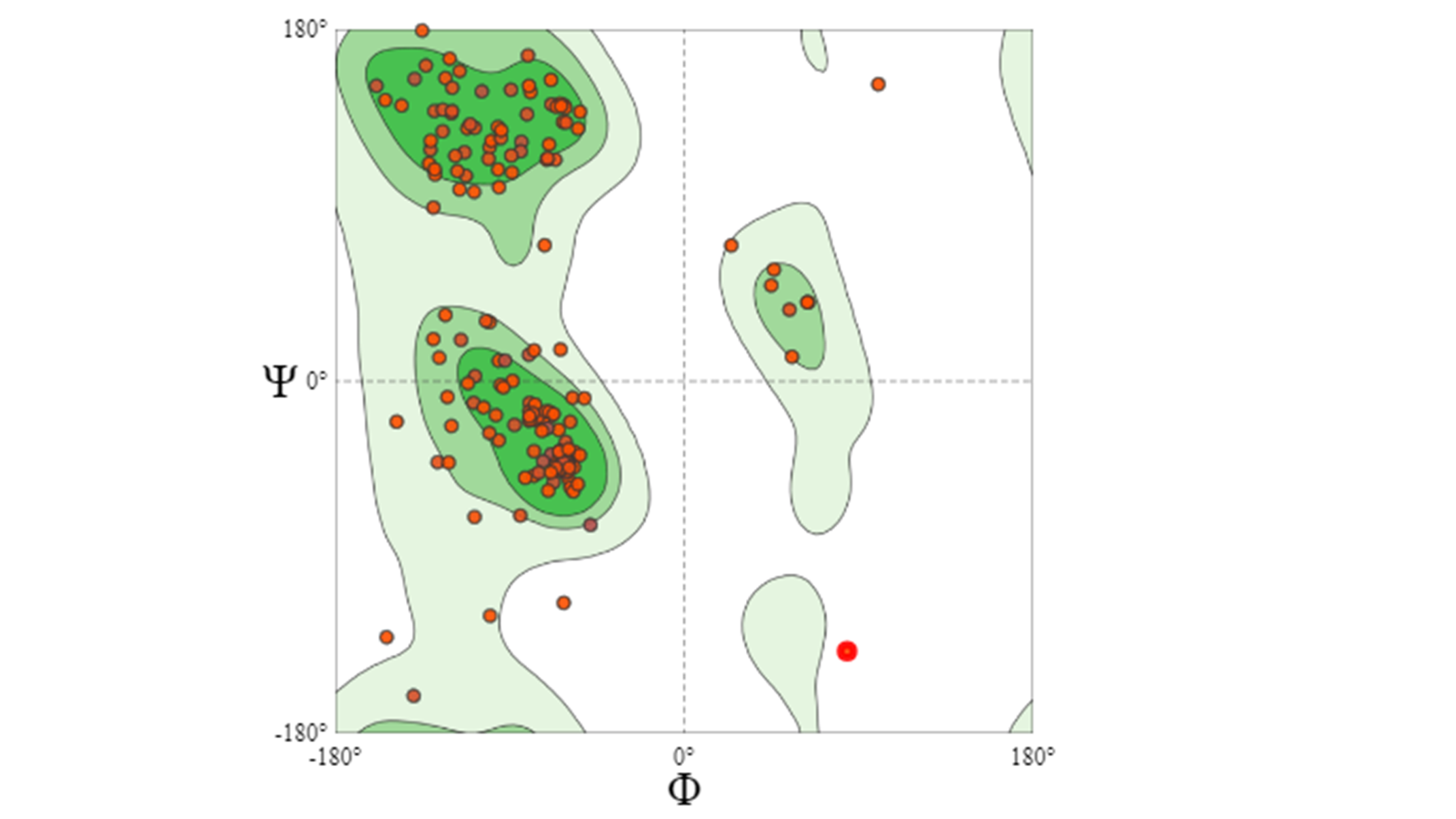


**Figure S1** **The structure validation of the R. oryzae RdRp model built in silico.** The scatter plot of Ψ (y-axis) versus Φ (x-axis) for each amino acid is represented by red circles (Ramachandran plot). The dark-green region represents the most favorable region, while green, faint-green, and white regions represent the allowed, strictly allowed, and forbidden regions.
